# Prospective assessment of the risk of obstructive sleep apnea in patients attending a tertiary health facility in Sub-Saharan Africa

**DOI:** 10.11604/pamj.2014.17.302.2898

**Published:** 2014-04-21

**Authors:** Obianuju Beatrice Ozoh, Njideka Ulunma Okubadejo, Ayesha Omolara Akinkugbe, Oluwadamilola Omolara Ojo, Chinyere Nkiru Asoegwu, Casmir Amadi, Ifedayo Odeniyi, Amam Chinyere Mbakwem

**Affiliations:** 1 Departments of Medicine, Faculty of Clinical Sciences, College of Medicine, University of Lagos, Lagos State, Nigeria; 2 Departments Surgery, Faculty of Clinical Sciences, College of Medicine, University of Lagos, Lagos State, Nigeria

**Keywords:** Obstructive sleep apnea, excessive day time sleepiness, tertiary hospital, Nigeria

## Abstract

**Introduction:**

The impact of Obstructive sleep apnea (OSA) in worsening outcomes is profound, especially in the presence of comorbid conditions. This study aimed to describe the proportion of patients at a high risk of OSA in our practice setting.

**Methods:**

The STOP BANG questionnaire and the Epworth Sleepiness scale were used to assess for OSA risk and excessive daytime sleepiness respectively. Hospitalized patients and out-patients were recruited. Intergroup differences in continuous variables were compared using the analysis of variance. The proportion of patients with high risk of OSA and excessive daytime sleepiness was presented as frequencies and group differences compared with the Pearson χ^2^ test. Independent risk predictors for OSA were assessed in multivariate logistic regression analysis.

**Results:**

A total of 1100 patients (53.4% females) participated in the study. Three hundred and ninety nine (36.3%) had a high risk of OSA, and 268 (24.4%) had excessive daytime sleepiness. Of the participants with high OSA risk, 138 (34.6%) had excessive daytime sleepiness compared to 130 (18.5%) of those with low OSA risk (p).

**Conclusion:**

A significant proportion of patients attending our tertiary care center are at high risk of OSA.

## Introduction

Obstructive sleep apnea (OSA) is a form of sleep disordered breathing characterized by repetitive partial or complete upper airway obstruction during sleep resulting in episodes of hypoxia and frequent arousals [1]. The adverse consequences include excessive daytime sleepiness, impaired cognition, and reduced vigilance with higher risk for motor vehicular and occupational accidents. There is a strong association of OSA with adverse cardiovascular events like stroke, coronary artery disease, diabetes mellitus, and cardiac arrhythmias as well as an overall impairment in the health related quality of life [2–5].

There is a wide variation in published prevalence of OSA (assessed by polysomnography) ranging from 2 to 30%, presumably consequent upon varying methodologies including sampling schemes, instrument and technique used for monitoring sleep and breathing, diagnostic criteria and population studied [6–11]. The prevalence of a high risk of OSA using screening questionnaires ranges from 20 - 80%, with higher rates from hospital-based compared to population-studies [12–14].

The strongest risk association for OSA is with obesity (including central obesity) [6, 11]. Other risk factors for OSA include male gender, menopause, increasing age (with a plateau at 65 years), and altered craniofacial anatomy such as retrognathia, tonsilar hypertrophy, enlarged tongue or soft palate, inferiorly positioned hyphoid bone, maxillary and mandibular retroposition, and decreased posterior airway space [8–11, 15, 16]. Cigarette smoking (including second-hand smoke), polycystic ovarian syndrome, hypothyroidism and pregnancy have also been implicated as risk factors [17–20]. Large scale studies have confirmed a role for inheritance and familial factors in the genesis of OSA [21–23].

The gold standard for diagnosis of OSA is laboratory polysomnography (PSG) however, polysomnography is expensive and not readily available, and only approximately 10% of the demand for PSG testing in patients with suspected OSA is met [24, 25]. The diagnosis of OSA however can be enhanced by use of validated questionnaires to identify those at high risk for further assessment [26, 27].

There is limited data on the burden of OSA in Nigeria. Available data reports a 20% prevalence of high risk OSA among hospital workers [12]. This risk is expected to be higher in a hospital based population due to the presence of comorbid conditions. Increased awareness, early diagnosis and appropriate intervention are particularly important in persons at high risk especially in the presence of co-morbidities including medical and surgical conditions. The burden of OSA in such patients in the Nigerian context is unclear. This study was therefore designed to screen for the risk of OSA and daytime sleepiness in both hospitalized patients and outpatients attending the Lagos University Teaching Hospital (LUTH) Lagos, Nigeria. This study will improve our appreciation of the burden of OSA in Nigerian patients and provide data for comparison with other populations.

## Methods

### Study Description

This was a descriptive cross sectional study conducted over a 12 week period. The study protocol was approved by the Lagos University Teaching Hospital Health Research Ethics Committee (LUTHHREC) (ADM/DCST/HREC/185), a multi-specialty tertiary care facility in Lagos State, south western Nigeria. The study recruited adult patients (aged ≥ 15 years) from the inpatient and outpatient services of the LUTH. Recruitment in the inpatient setting was from the adult surgical and medical wards and included all patients hospitalized over a 2-week period. Ambulatory outpatient recruitments were from one surgical clinic (otorhinolaryngology) and six medical clinics (respiratory, neurology, endocrinology/diabetology, cardiology, nephrology, and dermatology). Informed consent was obtained from all patients or their proxies.

### Study instrument and administration

Demographic and clinical information were obtained from patients and their case records, and included the age, gender, tentative or final diagnosis, and presence of cardiovascular risk factors based on historical, prior diagnostic evaluation, or physical examination. Specifically, the presence any of the following - diabetes mellitus, hypertension, current cigarette smoking, obesity, and central adiposity, were documented, with patient's having any of these categorized as high cardiovascular risk. Standard current diagnostic criteria for the preceding conditions were employed [28–31]. Resistant hypertension was defined as the presence of uncontrolled hypertension despite the use of maximal doses of three antihypertensive drugs, one of which included a diuretic [32].

To assess the risk of OSA and daytime sleepiness, we employed the STOP-BANG questionnaire and Epworth Sleepiness Scale (ESS) respectively. All questionnaires were interviewer administered, and all interviewers were physicians trained in the administration of the questionnaires as part of the study protocol.

The STOP-BANG questionnaire is a simple validated 8 item instrument that asks about symptoms of Snoring, Tiredness, Observed apnea and a history of high blood Pressure [26, 33]. It also includes a section to document the body mass index, age, neck circumference, and gender. The STOP-BANG questionnaire was initially developed after factor analysis and reliability check in a cohort of 2467 patients of whom 211 underwent polysomnography for validation. The sensitivity of the STOP-BANG questionnaire in accurately diagnosing OSA in persons with AHI index greater than 5, 15 and 30 was 83.6, 92.9 and 100% respectively [26]. There was no significant difference between the sensitivities of the STOP-BANG, Berlin questionnaire and the American Society of Anaesthesiology checklist for OSA screening as demonstrated among 117 patients who underwent polysomnography (65.6-75.9%, 68.9-87.2%, and 72.1-87.2% respectively) [33]. A systematic review of the various screening tools for OSA recommends using the STOP-BANG questionnaire because it has higher methodological quality and easy to use features [34]. To use the STOP-BANG questionnaire, a total score of 3 and above is considered a high risk of OSA [33].

The Epworth Sleepiness Scale© (ESS ©MW Johns 1990-1997) used with permission, is a validated 8-item questionnaire that measures the ease of falling asleep in the daytime under various circumstances as a measure of daytime hypersomlonence [35]. In the setting of OSA, the ESS has been shown to distinguish between patients with primary snoring and OSA [36]. A total score of 10 and above is suggestive of a sleep disorder possibly OSA [35].

### Data analysis

Data obtained was analyzed using the Statistical Software for Social Sciences (SPSS) version 17. Continuous variables are expressed as means and standard deviation, and intergroup differences compared using Analysis of variance (ANOVA). Group differences in discrete variables (presented as frequencies), are compared with the Pearson chi square. Independent risk predictors for OSA were assessed in multivariate logistic regression analysis. A p value of

## Results

### Baseline characteristics of study participants

The baseline data of the 1100 patients studied is shown in Table 1, and includes the gender distribution, age parameters, location of participants (in-patient or out-patient), specialty category (medical or surgical), anthropometric indices (body mass index, neck circumference, waist circumference). Hypertension was present in 456(41.5%) and 84(7.6%) had resistant hypertension. One hundred and nineteen (10.8%) had diabetes and 58(5.3%) were current smokers. However, the overall cardiovascular risk was high in 685 (62.3%).

**Table 1:** Baseline data and test scores of participants

Parameter	Description
Male: female ratio, *N(%)*	586 (53.4%): 514 (46.6%)
Mean age ± SD, *years*	43.9±16.1
Mean BMI ± SD, *kg/m* ^*2*^	25.3±5.7
Mean neck circumference ±SD, *cm*	35.3±3.3
Mean waist circumference ± SD, *cm*	90.3±13.4
Mean systolic blood pressure (mm/hg)	123.0±20.3
Mean diastolic blood pressure (mm/hg)	78.0±12.55
In-patient: out-patient ratio, *N (%)*	139 (12.6%): 961 (87.4%)
Medical patients: surgical patients, *N (%)*	150 (13.6%): 950 (86.4%)
STOP-Bang score (median, (IQR)	2, (1-3)
Proportion high risk OSA *N (%)*	399 (36.3%)
Epworth Sleepiness Scale score (median, (IQR)	6, (3-9)
Proportion with excessive daytime sleepiness (overall) *N (%)*	268 (24.4%)
Proportion with excessive daytime sleepiness (high risk OSA) *N (%)*	138 (51.5%)

SD= standard deviation; IQR= interquartile range; cm= centimeters

### Risk of obstructive sleep apnea and determinants in study participants

The risk of OSA (based on the STOP-BANG questionnaire) was low in 701 (63.7%) and high in 399 (36.3%) of the participants. None of the patients had a previous diagnosis of OSA. The proportion of medical patients with a high risk of OSA was significantly higher (361, 38%) compared to surgical patients (38, 25.3%) (P = 0.003). Risk of OSA was high in 59 (42.4%) inpatients and 340 (35.3%) outpatients (P = 0.1)

An initial univariate logistic regression analysis showed that all the variables explored (age, diabetes, resistant hypertension, cigarette smoking, abdominal adiposity and ESS score) were significantly associated with high risk of OSA (PTable 2). Age above 65 years, excessive daytime sleepiness (ESS score ≥ 10), presence of abdominal adiposity, resistant hypertension and high overall cardiovascular risk were independent determinants of a high risk of OSA. The magnitude of risk associated with these independent predictors of high risk (represented by the Odds ratio) was highest for persons aged above 65 years, those with excessive day time sleepiness, and presence of abdominal adiposity (Table 2)

**Table 2:** Determinants of high risk for obstructive sleep apnea

Variables	Standardized coefficient β	Standard error	95% Confidence Interval (SE)	P value	Odds Ratio (95% confidence interval)
Age above 65	-1.55	0.26	2.82 – 7.89	<0.0001	6.96 (4.33 – 11.21)
High ESS risk	-0.75	0.17	1.53 – 2.92	<0.0001	2.32 (1.75 – 3.08)
Abdominal adiposity	0.53	0.17	0.42 – 0.82	0.002	1.90 (1.47 – 2.44)
Diabetes	0.28	0.22	0.49 – 1.17	0.208	0.35 (0.24 – 0.52)
Cigarette smoking	0.52	0.31	0.32 – 1.09	0.094	0.26 (0.15 – 0.45)
Resistant hypertension	1.16	0.28	0.18 – 0.54	<0.0001	0.17 (0.10 – 0.28)
Cardiovascular risk	2.14	0.22	0.08 – 0.18	<0.0001	0.11 (0.08 – 0.16)

Multiple logistic regression analysis and determination of odds ratio. High risk for OSA was defined by STOP-Bang score of 3 and above (1 score assigned for any of the following present: age above 50 years, male gender, presence of hypertension, or obesity, or nuchal obesity).

### Relationship between OSA risk and excessive daytime sleepiness

**Figure 1:**
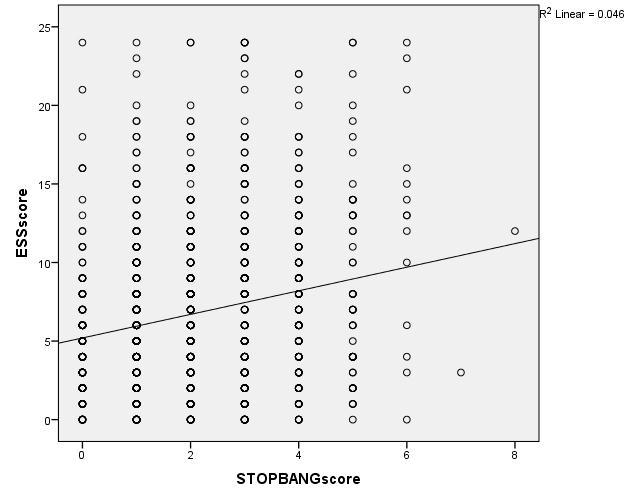
Relationship between STOP-bang score and Epworth Sleepiness Scale score

Based on the ESS score, 268 (24.4%) of the participants had excessive daytime sleepiness. Figure 1 shows a positive correlation between the STOP-BANG scores and the ESS scores. Of the participants with high risk of OSA, 138 (34.6%) also had excessive daytime sleepiness compared to 130 (18.5%) of those with low risk of OSA with excessive daytime sleepiness (p

## Discussion

Under-recognition of obstructive sleep apnea (OSA) worsens co-morbid cardiovascular diseases and impairs global quality of life, both cognitive and physical. The main finding in this study is that a significant proportion (36%) of patients attending our tertiary care hospital for diverse medical and surgical conditions is at a high risk of OSA. Other important findings are that those at high risk of OSA also have a high risk of excessive daytime sleepiness and that presence of abdominal adiposity and age above 65 years confer an increase in OSA risk, thus highlighting specific subgroups of patients who require formal diagnostic evaluation using polysomnography.

The prevalence of high risk of OSA in this study is comparable to that in earlier studies from other populations [10, 12–13]. Interestingly, none of the patients in our study had a prior diagnosis of OSA or was on treatment. This high rate of under-diagnosis in our practice setting possibly represents a low index of suspicion or physician unawareness of the burden of the disease, risk factors and the health hazards of untreated OSA. Among surgical patients, untreated OSA increases the risk of perioperative complications including difficult intubation, post-operative respiratory and cardiovascular complications, increased admission to the intensive care unit, prolonged hospital stay, and death [37, 38]. OSA also increases cardiovascular risk, as repeated episodes of apnea during sleep result in hypoxia, hypercapnia, raised intra-thoracic pressures, repeated arousals and sleep deprivation leading to sympathetic activation, metabolic dysregulation, endothelial dysfunction, systemic inflammation, hypercoagulability and left atrial enlargement [2]. Therefore untreated OSA worsens outcome in a wide range of medical conditions including hypertension, heart failure, cardiac arrhythmias, renal disease, stroke, myocardial infarction, asthma, epilepsy, reactive bladder, and urinary incontinence [2–4, 39–41].

Our findings corroborate the increased risk and prevalence of OSA with advancing age [8–11]. We found that age above 65 years was associated with the highest risk for OSA (Odds ratio 6.96) implying a further increase in risk of adverse cardiovascular outcomes in the elderly who often have other comorbid conditions. The mechanisms for the age related increase in the prevalence of OSA include increased deposition of fat in the pharyngeal area, lengthening of the soft palate, and changes in body structure surrounding the pharynx [42, 43].

In our study, high risk of OSA was associated with a high risk of excessive daytime sleepiness similar to findings in earlier reports of excessive daytime sleepiness as a major effect of OSA [44]. Excessive daytime sleepiness occurs due to lack of restful sleep and is of major public health concern as it increases the risk of road traffic accidents and other occupational injuries [5]. Also, there is an overall reduction in productivity arising from impaired neurocognitive functioning leading to poor concentration, depression, occupational difficulties, poor libido and overall negative impact on the quality of life [45, 46].

Our study demonstrates that OSA is a common co-morbidity in many hospital patients and therefore highlights the need to identify and treat these patients. Treatment strategies in OSA are specific to the individual scenario, but may include weight loss (which improves overall cardiovascular risk), use of oral appliances that keep the airway open during sleep, surgery (uvulopalatopharyngoplasty, tonsillectomy, radiofrequency ablation of the base of the tongue, mandibular advancement), and the use of continuous positive airway pressure (CPAP) [44, 47–50]. CPAP has been shown to be the most effective treatment in controlling symptoms and improving cardiovascular risk [50]. Smoking cessation, avoiding alcohol, sedatives and sleeping pills in the evenings are adjunctive modalities that improve symptoms [47].

The findings in this study are limited by some factors identified here. Risk assessment of OSA using questionnaires is not diagnostic but identifies persons at high risk in whom further evaluation by polysomnography is warranted. The STOP-BANG questionnaire though previously validated was not re-validated in our patient population using polysomnography, however, the format of the questions (yes/no) and the standardization of the measurements make its use as a screening tool generalizable. This study was tertiary hospital-based and the study population comprised of a heterogeneous group of patients with potential over-representation of more severely ill persons and those with clinical conditions that independently increase OSA risk (cardiovascular, oto-rhinolaryngology). However it does provide data on those at highest risk of adverse outcome. Community-based evaluation would have its own benefit of defining the risk burden in the general population and for comparison with other populations.

## Conclusion

In conclusion, OSA is a prevalent but under-diagnosed condition in patients attending our tertiary care center. Additional risk factors observed include increasing age (above 65 years) and abdominal adiposity. Excessive daytime sleepiness commonly occurs in those at high risk of OSA putting them at risk of accidents and neuro-cognitive impairment in the daytime. Increased awareness of OSA among health care providers and patients will improve overall patient care and disease outcome and ultimately reduce mortality. Despite limited resources and difficulty in confirming the diagnosis of OSA as may occur in our setting, treatment of patients who are at high risk from screening and in whom symptoms are suggestive of OSA should be considered especially in the presence of comorbid conditions.
